# Genome wide identification of lncRNAs and circRNAs having regulatory role in fruit shelf life in health crop cucumber (*Cucumis sativus L.*)

**DOI:** 10.3389/fpls.2022.884476

**Published:** 2022-08-03

**Authors:** Shyam S. Dey, Parva Kumar Sharma, A. D. Munshi, Sarika Jaiswal, T. K. Behera, Khushboo Kumari, Boopalakrishnan G., Mir Asif Iquebal, R. C. Bhattacharya, Anil Rai, Dinesh Kumar

**Affiliations:** ^1^Division of Vegetable Science, ICAR-Indian Agricultural Research Institute, New Delhi, India; ^2^Centre for Agricultural Bioinformatics, ICAR-Indian Agricultural Statistics Research Institute, New Delhi, India; ^3^ICAR-National Institute of Plant Biotechnology, New Delhi, India

**Keywords:** cucumber, lncRNA, circRNA, regulatory role, fruit firmness, shelf life

## Abstract

Cucumber is an extremely perishable vegetable; however, under room conditions, the fruits become unfit for consumption 2–3 days after harvesting. One natural variant, DC-48 with an extended shelf-life was identified, fruits of which can be stored up to 10–15 days under room temperature. The genes involved in this economically important trait are regulated by non-coding RNAs. The study aims to identify the long non-coding RNAs (lncRNAs) and circular RNAs (circRNAs) by taking two contrasting genotypes, DC-48 and DC-83, at two different fruit developmental stages. The upper epidermis of the fruits was collected at 5 days and 10 days after pollination (DAP) for high throughput RNA sequencing. The differential expression analysis was performed to identify differentially expressed (DE) lncRNAs and circRNAs along with the network analysis of lncRNA, miRNA, circRNA, and mRNA interactions. A total of 97 DElncRNAs were identified where 18 were common under both the developmental stages (8 down regulated and 10 upregulated). Based on the back-spliced reads, 238 circRNAs were found to be distributed uniformly throughout the cucumber genomes with the highest numbers (71) in chromosome 4. The majority of the circRNAs (49%) were exonic in origin followed by inter-genic (47%) and intronic (4%) origin. The genes related to fruit firmness, namely, polygalacturonase, expansin, pectate lyase, and xyloglucan glycosyltransferase were present in the target sites and co-localized networks indicating the role of the lncRNA and circRNAs in their regulation. Genes related to fruit ripening, *namely*, trehalose-6-phosphate synthase, squamosa promoter binding protein, WRKY domain transcription factors, MADS box proteins, abscisic stress ripening inhibitors, and different classes of heat shock proteins (HSPs) were also found to be regulated by the identified lncRNA and circRNAs. Besides, ethylene biosynthesis and chlorophyll metabolisms were also found to be regulated by DElncRNAs and circRNAs. A total of 17 transcripts were also successfully validated through RT PCR data. These results would help the breeders to identify the complex molecular network and regulatory role of the lncRNAs and circRNAs in determining the shelf-life of cucumbers.

## Introduction

In recent times, functions of a class of RNAs with little or no protein coding potential is an area of interest with the advancement and wide application of next-generation sequencing technologies (Liu et al., 2015). Recent advancements in high throughput RNA-sequencing technologies have played a key role in the identification and understanding of the role of several groups of novel non-coding RNAs (ncRNA) in different organisms including plants (Ravasi et al., 2006). The upcoming new edge technologies in next-generation sequencing along with the bioinformatics tools have revolutionized the field of biological sciences to understand and explore the functions of all the important groups involved in genes at transcription, post-transcription, post-translation, and even epigenetic levels (Li et al., 2018). Novel long non-coding RNAs (lncRNAs) were discovered and advocated as one of the emerging players to understand important biological processes, growth, and development besides stress response (Yu et al., 2019; Budak et al., 2020; Jha et al., 2020). LncRNA with a length of more than 200 nt is reported to be involved in a wide variety of processes such as splicing, gene inactivation, and translation (Costa, 2005; Liu et al., 2013; Ma et al., 2013). LncRNAs are transcribed by RNA polymerase II or III, and additionally, by polymerase IV/V in plants (Dinger et al., 2008, 2009; Wierzbicki et al., 2008). LncRNAs act as the largest class of diverse RNAs as ‘biological regulators’ with a regulatory role in transcription and post-transcription levels (Bohmdorfer and Wierzbicki, 2015; Chekanova, 2015). The regulatory role of the lncRNAs in an array of biological processes in plants has been elaborated by different research groups (Franco-Zorrilla et al., 2007; Nejat and Mantri, 2018; Shin et al., 2018; Corona-Gomez et al., 2020; Sun Z. et al., 2020).

Besides lncRNAs, another class of ncRNAs called circular RNAs (circRNAs) has been projected as one of the important players in regulating biological processes through transcription and genome imprinting. The first circRNA was identified long back by Sanger et al. (1976) and was advocated as the byproduct of abnormal splicing with negligible functional potential. Back-splicing of exons from precursor mRNA is responsible for the production of circRNAs. RNA circle is formed with the connection of the downstream 5′ splice site with the upstream 3′ splice site and ligation by a 3′–5′ phosphodiester bond at the site of connection (Chen, 2016). Characterization of the circRNA elucidates that they can be originated from intron, exons, and intergenic regions and their expression pattern is specific to cells, tissues, and developmental stages. Because of the higher stability of the circRNA in comparison to the conventional RNAs, they are more likely to be involved in various biological processes (Li et al., 2017).

Cucumber (*Cucumis sativus* L.) is one of the most important vegetable crops cultivated worldwide and serves as a model plant species for genetic and genomic studies because of its rich source of genomic information. It is known for its wider therapeutic and pharmacological applications. It is used as an antidiabetic having lipid lowering and antioxidant properties (Mukherjee et al., 2013). Smaller genome size, distinct sex expression, and worldwide distribution facilitate detailed genomics studies for several complex mechanisms regulating different biological processes in cucumber. Shelf-life is an important trait determining storability and transport of highly perishable crops like most the vegetables. The various measures widely adopted to improve the shelf life of the harvested produce in the high-income economies are not popular in low-income developing countries because the high-cost intervention affects the final retail price of the fresh produce (Friedman et al., 2020). It is estimated that about 30% of the fresh fruits harvested are lost because of post-harvest deterioration, ripening, and decay (Gajanana et al., 2011; Kasso and Bekele, 2018). Therefore, reducing the post-harvest loss of perishable crops like fruits and vegetables is a global mission for food availability and reducing hunger (Porat et al., 2018). Among the vegetable crops, fruit ripening and shelf-life are better understood in the climacteric crop, tomato through detailed translational studies. However, ripening, shelf-life, and biological processes involved in post-harvest decay are poorly understood in a model non-climacteric crop, like cucumber. A natural variant, DC-48 with extended shelf-life was isolated at the Division of Vegetable Science, ICAR-Indian Agricultural Research Institute, New Delhi (28.6377° N, 77.1571° E). This genotype can be stored under room condition without any low temperature storage up to 10 days after harvesting without loss of the fresh green color and fruit firmness. However, the detailed physiological, biochemical, and molecular networks associated with the extended shelf-life of DC-48 are yet to be explored. Roles of lncRNA and circRNA associated with this extremely important trait in the genotype, DC-48 will provide insight into the complex mechanisms associated with the extended shelf-life. Another contrasting genotype, DC-83 with very poor shelf life and become unfit for marketing within 2–3 days after harvesting (Pradeepkumara et al., 2022) was taken along with DC-48 for the present study to understand the role of lncRNA and circRNA in regulating shelf-life of cucumber. In most vegetable crops, it is widely known that pre-harvest metabolic processes driven by the genetic makeup of the mother plant play important role in determining the post-harvest behavior of the fruits (Weston and Barth, 1997; Arah et al., 2015). It was also evident from studies in a related crop such as in the case of melons that early fruit developmental stages are critical in determining the post-harvest physiology of the fruits (Saladié et al., 2015). Most cucumber fruits attain harvestable maturity at 10 days after pollination (DAP). Therefore, it would be ideal to conduct RNAseq analysis one at the early stage of development and another at harvesting maturity.

In cucumber differentially expressed lncRNA and circRNA in response to heat stress were identified by He et al. (2020) who revealed the role of these groups of novel ncRNAs in stress response. Besides, the role of lncRNA and miRNA were identified in cucumber in response to long term waterlogging conditions (Kȩska et al., 2021). In watermelon, the pivotal role of lncRNAs and circRNA in defense response in relation to cucumber green mottle mosaic virus has been reported by Sun Y. et al. (2020). Genome wide identification of lncRNA and circRNA for fruit ripening has been reported in a number of crops like *C. melo* (Tian et al., 2019) and strawberry (Tang et al., 2021). However, to the best of our knowledge, identification of lncRNAs and circRNAs in relation to the extended shelf-life in cucumber is still warranted. Therefore, this study was conducted to identify and understand the role of lncRNA and circRNA and complex molecular networks in relation to the extended shelf life in two contrasting cucumber genotypes, namely DC-48 and DC-83.

## Materials and methods

### Plant material and sample preparation

Two contrasting genotypes, DC-48 (better keeping quality) and DC-83 (poor keeping quality) were grown in the research field of the Division of Vegetable Science, ICAR-Indian Agricultural Research Institute, New Delhi (28.6377° N, 77.1571° E) under protected condition using insect proof net during the spring summer season of 2019. The genotype, DC-48 and DC-83, were slicing type cucumbers and belong to the same species, *C. sativus* L. Description of the genotypes and their detailed characterization for keeping quality is discussed in our earlier report (Pradeepkumara et al., 2022). Suitable cultural practices were followed for raising a healthy crop. The fully developed fruits at different developmental stages after pollination from the healthy plants were used for determining the shelf life and whole genome RNA-sequencing. Samples were collected at 5DAP and 10DAP using the normally developed fruits for RNA-seq. Sampling of the fruits and isolation of RNA was done in triplicate as per the procedure discussed by Pradeepkumara et al. (2022). Uniformly peeled surface tissues of the fruit epicarp (2 mm) were used for RNA extraction and high throughput sequencing. Post-harvest biology of the cucumber fruits is predominantly determined by the epicarp structure and texture, therefore, considered ideal tissues for RNA-seq analysis. TRIzol reagent (Invitrogen, Waltham, MA, United States) was used for the extraction of total RNA and the concentration and quality of the extracted RNA were determined using Bio-analyzer (Agilent, United Kingdom). Whole genome RNA sequencing was performed using 100 ng μl^–1^ extracted RNA (Illumina HiSeq X10). For the quantitative real-time polymerase chain reaction (qPCR) analysis, RNA was treated with DNaseI from the DNA-free kit (Promega Corporation, United States) and then checked by PCR to ensure that there was no contaminating DNA. First-strand cDNA was synthesized using the High-Capacity cDNA Reverse Transcription Kit as per the manufacturer’s protocol (Promega Corporation, Madison, WI, United States).

### Data pre-processing and assembly

The paired-end HiSeq Illumina reads of 2*151 bp, generated from two contrasting genotypes of cucumber, i.e., DC-48 (extended shelf life; label:1) and DC-83 (poor shelf life; label:2), at two developmental stages *viz*., 5 DAP (Days after pollination (labelled as A) and 10 DAP (labeled as B) in triplicates were pre-processed. The read quality was checked using the tools fastQC^1^. The pre-processing of raw reads was done using *Trimmomatic* v0.39 tool (Bolger et al., 2014) to remove low-quality reads and adapter sequences. The filters used were read length ≤ 36, poor quality ≤ 3 and HEADCROP: 10 bases. After pre-processing, the high-quality reads were used for the construction of a *de-novo* transcriptome assembly using *Trinity* software (Haas et al., 2013) resulting in 186,184 transcripts.

### Identification of novel long non-coding RNAs

For the identification of lncRNA, transcripts longer than 200 base pair were considered. We found all the 186,184 transcripts to be above 200 base pair in length. Further, the transcripts with FPKM values greater than 0.5 were filtered. Studies have shown that lncRNAs in general have lower quality and shorter ORFs than the protein coding mRNAs. The ORFs in each transcript were predicted with an ORF finder^2^ and those having ORFs longer than 300 nucleotides (or 100 amino acids) were removed as lncRNAs have shorter ORFs than the protein coding mRNAs. This was followed by Blast2Go (Conesa and Götz, 2008) in order to filter and remove the already annotated sequences. A search against Pfam (Mistry et al., 2021) database was done to identify the protein families if any. Further, the non-coding transcripts were filtered after processing through TransDecoder ver. 5.5.0^3^ categorized the transcripts into coding and non-coding. Binary classifiers like CPC2 ver. 1.0.1 (Kang et al., 2017) and PLEK ver. 1.2 (Li et al., 2014) were employed further to classify the remaining sequences into coding (score > 0) or non-coding (score < 0) based on the scores. In order to remove transcripts having certain housekeeping RNAs, namely, rRNAs, tRNAs, snoRNAs, and other ncRNAs, a BLAST search against the RNA Central database^4^ was made, and removed the transcripts showing ≥ 95% identity and ≤ 3 mismatches to retain the lncRNAs.

### Comparison of identified lncRNAs With known lncRNAs

The identified lncRNAs in the study were compared with the available plant lncRNAs in different databases like CANTATAdb and PLncDB. LncRNAs are known to be poorly conserved as compared to protein coding mRNAs across species. The lncRNAs from 39 plant species available at CANTATAdb (Version 2.0)^5^ were downloaded. Local blast was performed against the identified cucumber lncRNAs at cutoff of > 70% identity. Similarly, our identified lncRNAs were local Blast with the cucumber lncRNAs from PLncDB Version 2.0^6^ and sequences with 100% identity were reported as a significant match.

### Identification of differentially expressed lncRNAs (DElncRNAs)

The DElncRNAs were identified from all the four comparison sets (1A:1B, 1A:2A, 1B:2B, and 2A:2B). The assembled transcripts obtained after reads’ pre-processing were aligned with the paired-end reads using Bowtie2 (Langmead and Salzberg, 2012). RSEM (RNA-Seq by expectation maximization) was used to measure the transcript abundance level of genes and isoforms for each set and calculate the expression (Li and Dewey, 2011). EdgeR (Empirical Analysis of Digital Gene Expression in R) was used for expression analysis of transcripts from inter-varietal and intra-varietal developmental stages at stringent parameters (FDR < 0.001, *p* < 0.05 and log2fold change = ± 1) (Robinson et al., 2010). The differentially expressed genes (DEGs) obtained from the assembled transcripts were further compared with the identified lncRNA to find the DElncRNA.

### Identification of the lncRNAs interacting miRNAs and their mRNA targets

In order to check if the identified lncRNAs could potentially be targeted by miRNAs or could be target mimicry for miRNAs, psRNATarget (Dai et al., 2018) was used. Here, lncRNA sequences were used as input for target candidates. Matches with expectation ≤ 3 was considered significant in our study. To predict endogenous target mimics (eTMs), TAPIR (Bonnet et al., 2010) was used with an MFE ratio ≥ 0.6 (Bouba et al., 2019).

For creating the lncRNA-miRNA-mRNA network, the mRNA targets of the identified miRNAs were predicted. For this, psRNATarget was run with identified miRNA sequences and cucumber cDNA sequences available at the server as input. Matches with expectation ≤ 3 were considered significant.

### Identification of mRNA targets of DElncRNA

LncRNAs can target mRNA and affect gene expression. Both, *cis* and *trans* targets were identified in the cucumber reference genome. *Cis*-targets were identified by searching the 100kb window upstream and downstream of the identified lncRNAs using the window-bed option of Bedtools. The *trans* mRNA targets for all the differential expressed lncRNA were identified using lncTAR (Li et al., 2015) using cucumber cDNA sequences^7^. A high normalized deltaG (nDG) threshold (-0.20) was set to get high confidence lncRNA-mRNA interacting pair.

### Identification of circular RNA and differentially expressed CircRNA (DEcircRNAs)

To identify the circRNAs, the high-quality clean reads obtained after pre-processing were used. These reads were aligned against the *C. sativus* reference genome using BWA (v0.7.17, mem-T 20) employing the circRNA identification tool, CIRI2 (v2.1.1) (Gao et al., 2018). The resulting output SAM file was further inspected by the CIRI2 core program to identify the putative circular RNA. The Differentially expressed circular RNA (DEcircRNAs) were obtained from all the four comparison sets (1A:1B, 1A:2A, 1B:2B, and 2A:2B) by comparing the identified circRNA to DEGs obtained from the assembled transcripts at parameters, i.e., FDR < 0.001, *p* < 0.05 and log2fold change = ± 1 (Robinson et al., 2010).

### Analysis of lncRNA, miRNA, circRNA, and mRNA network interactions

The roles of lncRNAs were studied by constructing a miRNA–lncRNA–mRNA network based on differentially expressed lncRNAs and miRNAs, and the target pairs of miRNAs–lncRNAs, miRNAs–mRNAs, lncRNAs–mRNAs and circRNA-miRNA. The regulatory networks contained miRNAs, lncRNAs acting as miRNA targets, mRNAs acting as lncRNA targets, and mRNAs acting as miRNA targets, and also circRNA acting as miRNA target. Cytoscape 3.7.2 software (Shannon et al., 2003) was used to visualize the regulatory networks of miRNA–lncRNA–mRNA.

### Quantitative real-time PCR validation of identified lncRNAs

A total of 17 differentially expressed transcripts were selected for validation using qRT-PCR at different developmental stages of the two contrasting genotypes. The primers were designed using IDT primer quest software^8^. cDNA was synthesized from DNase-treated total RNA (2 μg) using Go Script TM reverse transcription system kit (Promega, USA) as per the manufacturer’s instructions and diluted 20 times with nuclease free water. The qRT-PCR was performed on lightcycler 96 system Real-Time PCR (Roche, Indianapolis, IN, United States) in a final volume of 10 μl containing 1 μl diluted cDNA (200 ng), 5 μl 2xSYBR Green (Go Taq qPCR system, United States), 0.4 μl each of forward and reverse primer (10 μM), and 3.2 μl RNase-free water as per the manufacturer’s instructions with three biological replicates. The thermal cycling conditions were as follows: 95°C for 1 min followed by 40 repeated cycles of 95°C for 10 s, 58°C for 30 s, and 72°C for 30 s. Relative gene expression was determined using 2-ΔΔCT method by normalizing the Actin gene expression. The primers used for qRT-PCR validation along with the description were listed in Supplementary Table 1.

### Development of web genomic resources of cucumber lncRNAs

Long non-coding RNA *C. sativus* Extended shelf-life Database (LncR-CsExSLDb) is a “three-tier architecture” based relational database with client-, middle- and database tier. It catalogs the predicted lncRNAs, circular RNAs, DElncRNAs, DEcircRNAs, lncRNA targets of miRNAs, mRNA targets of miRNAs, mRNA targets of lncRNAs, and circRNA targets of miRNAs in cucumber (*C. sativus*) transcriptome. All the data has been stored in MySQL tables as a database tier. LncR-CsExSLDb provides various information, like differentially expressed lncRNA (DElncRNA), miRNA which could target the predicted lncRNA and mRNA targets of lncRNA. It also provides information on the miRNA targets in terms of mRNA. Users can also retrieve the predicted putative circular RNAs. miRNA that can possibly target circRNA are also listed. For database browsing, web pages were developed in html, along with CSS and javascript in the client tier. This web-genomic resource is freely available for academic use at http://webtom.cabgrid.res.in/lncrcsexsldb.

## Results

### Data pre-processing and assembly

Approximately 0.03% of the generated paired-end Illumina reads from the two contrasting cucumber varieties, DC-48 (extended shelf-life) and DC-83 (poor shelf-life) were dropped in all four sets of data, i.e., 1A, 1B, 2A, and 2B. The *de novo* assembly using the trinity of the pooled 147 MB clean reads yielded around 186K transcript having N50 2.9 kbp and GC content 38.89%. Almost 92% of these assembled transcripts were mapped on the available *C. sativus* genome^9^.

### Identification of novel long non-coding RNAs

All the assembled transcripts having a length > 200 base pairs were further filtered based on the removal of transcripts having FPKM ≥ 0.5, leading to the retention of 77223 transcripts. Further, after ORF prediction in all the transcripts using ORF Finder, the transcripts having ORF longer than 300 bp were removed leading to 41712 transcripts to be used for further analysis. After screening these sequences for similarity with already annotated sequences through BLAST2GO Pro ver. 3.1, a total of 23816 sequences were retained for further analysis, after discarding 17,896 significantly similar sequences. The output of the PFAM search was parsed through TransDecoder that identified a total of 351 transcripts with coding potentials, hence these were removed. The remaining 23,465 transcripts were analyzed for coding potentials using CPC2 and PLEK, which identified 2 and 173 of these having coding potentials, hence discarded. Finally, 23,290 non-coding transcripts were identified (Supplementary Table 2).

**FIGURE 1:**
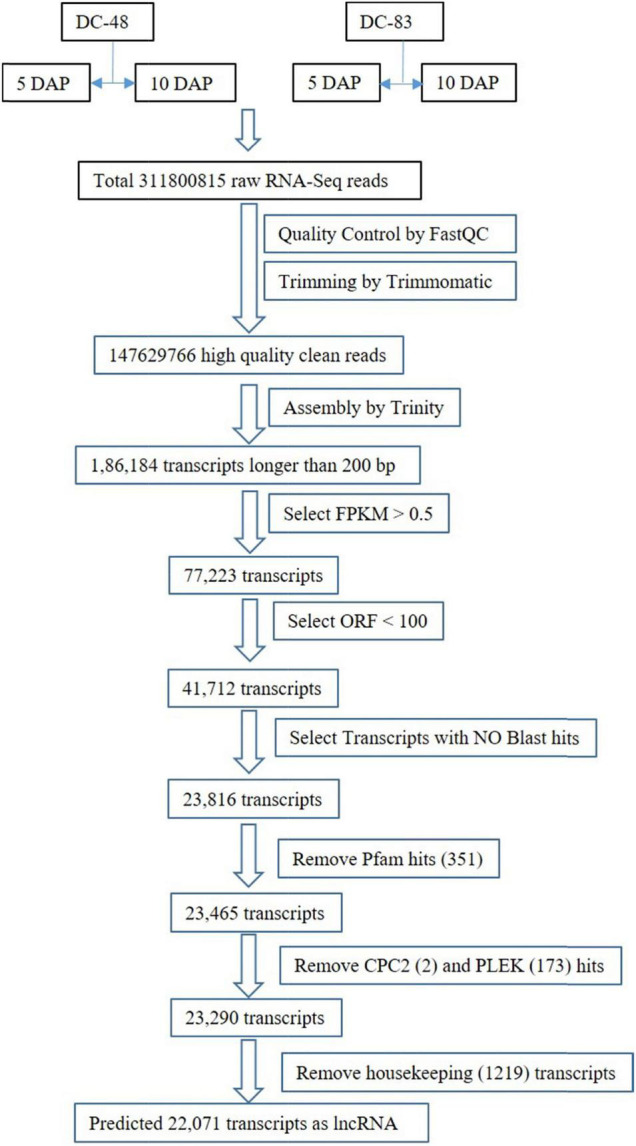
Work flow for identification of genome wide lncRNA from Cucumber transcriptome.

Housekeeping genes like tRNA, rRNA, and other non-coding RNAs were also removed. A sequence search against the RNA Central database identified 1219 housekeeping genes and were removed and all the matches with lncRNA were retained. After filtering, finally, a total of 22071 transcripts were retained for all the further analysis (Supplementary Table 3). The workflow of lncRNA prediction has been shown in Figure 1.

**FIGURE 2:**
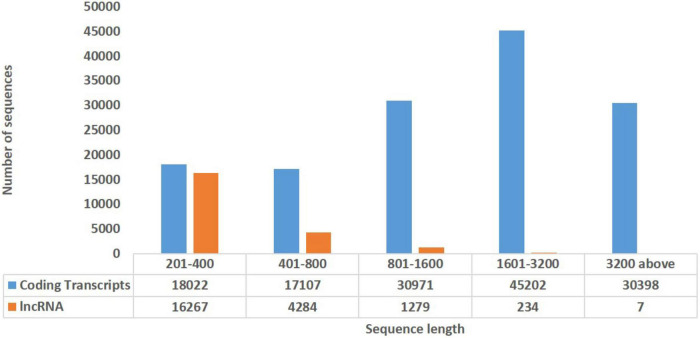
Distribution of sequences present in coding transcripts and lncRNA based on their length.

**FIGURE 3:**
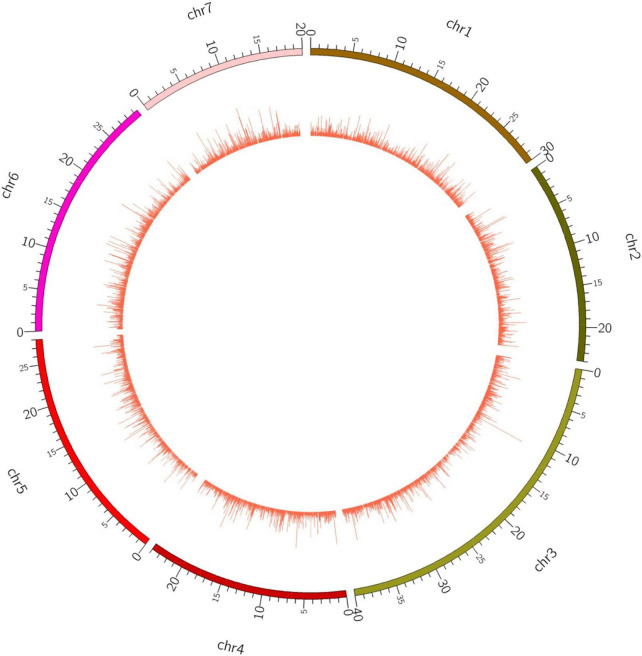
Chromosome-wise distribution of identified lncRNA on cucumber genome.

**FIGURE 4:**
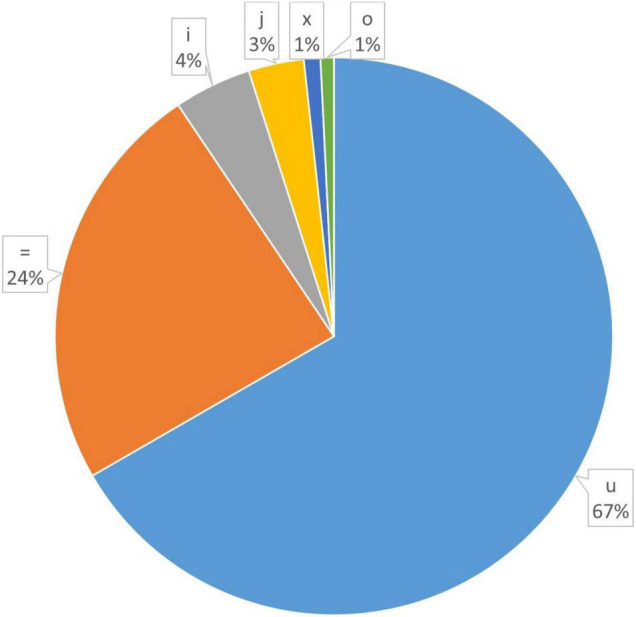
Classification of predicted lncRNAs based on their genomic locations.

In order to explore the trend of occurrence of lncRNAs in the cucumber genome, various characteristics of the predicted lncRNAs were examined. It was observed that the sequence length of the majority of the lncRNAs ranged between 201–400 (73.7%), followed by 400–800 (19.4%) while coding transcripts were abundant with the length ranging between 1600–3200 (Figure 2). The circos plot shows the distribution of the identified lncRNA on 7 reference cucumber chromosomes (Figure 3). The genomic locations of lncRNAs were classified into six categories with respect to the reference annotation. Most of the lncRNAs were in the unknown lncRNAs (u) category (∼67%), followed by complete intronic ( = ) (∼24%), fully in reference intronic (i) (∼4%), splice junction (j) (∼3.0%), exonic overlap other strands (x) (∼1%) and exonic overlap (o)(∼1%) (Figure 4).

### Comparison of identified lncRNAs with known lncRNAs

The blast search against CANTATAdb revealed 3782 unique matches representing conserved nature of these lncRNA across 39 plants species (Supplementary Table 4). Also, a total of 3408 unique matches were retrieved from PlncDB database, specific to cucumber lncRNAs. After removing the redundant sequences, a total of 5522 known lncRNA from CANTATAdb and PlncDB were achieved, while 16549 lncRNAs were identified as novel.

### Identification of differentially expressed lncRNAs (DElncRNAs)

**FIGURE 5:**
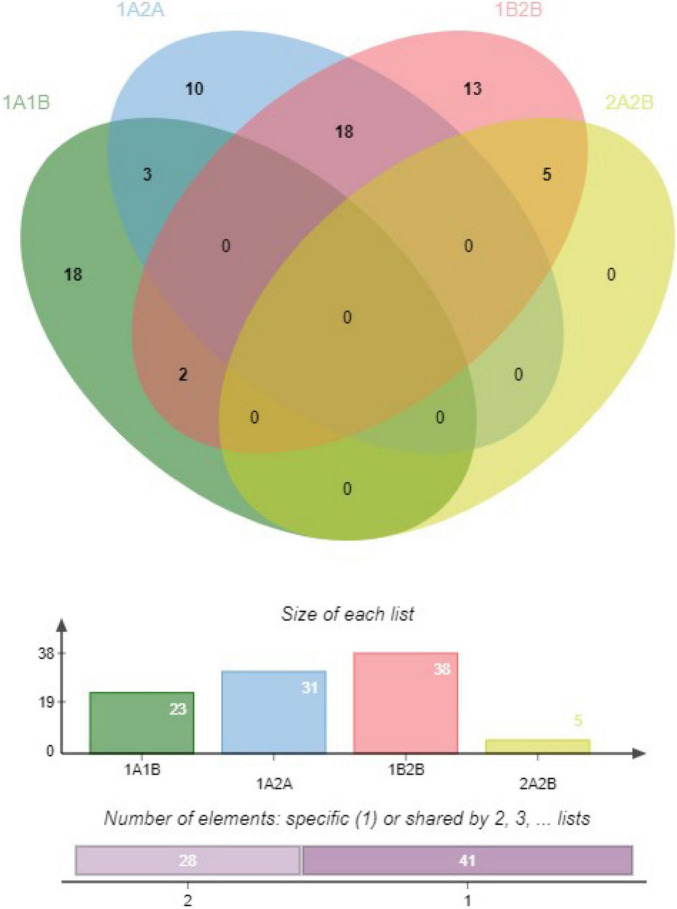
A venn diagram showing the distribution of differential expressed lncRNA in the four conditions that are compared in this study.

A total of 4188 unique DEGs were obtained from the four comparison sets (1909, 1624, 1712, and 189 in 1A:1B, 1A:2A, 1B:2B, and 2A:2B at defined cutoff, respectively using the EdgeR (Supplementary Table 5). These DEGs were further compared with the identified 22071 lncRNAs to filter DElncRNA. A total of 23, 31, 38, and 05 lncRNA were found to be significantly different in the four comparison sets, *namely*, 1A:1B, 1A:2A, 1B:2B, and 2A:2B, respectively (Table 1 and Supplementary Table 6). We found 10, 18, and 13 unique DElncRNAs in the 1A:2A, 1A:1B, and 1B:2B comparison. The shared and unique DElncRNAs are represented in the form of a Venn diagram (Figure 5). Hierarchical clustering of DElncRNAs, as well as samples based on transcripts abundance in the form of heatmap of the three biological replicates per sample, is represented in Supplementary Figure 1.

**TABLE 1:** Number of differentially expressed lncRNA in various comparison sets.

Comparison	Identified DElncRNAs	Upregulated lncRNAs	Downregulated lncRNAs
1A1B	23	6	17
1A2A	31	18	13
1B2B	38	21	17
2A2B	05	0	5

### Identification of the lncRNAs interacting miRNAs and their mRNA targets

We predicted 136 (99 unique) miRNAs which could target our identified DElncRNA using psRNATarget (Supplementary Table 7). Further, the target mRNA for these identified miRNAs were identified by psRNATarget by giving miRNA sequences as input and searching against the *C. sativus* cDNA library (ASM407v2) available at psRNATarget. This resulted in 2001 (1228 unique) mRNAs acting as targets for miRNAs (Supplementary Table 7). Moreover, we found that 16 lncRNAs could act as endogenous target mimics (eTMs) for 15 miRNAs, making 23 possible combinations (Table 2).

**TABLE 2:** Complementary pairing of lncRNAs targeted by miRNAs in cucumber.

Target	MiRNA	MFE ratio	Alignment
TRINITY_DN14071_c2_g1_i6	ath-miR5021	0.94	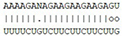
TRINITY_DN14588_c1_g3_i10	sbi-miR1435a	0.92	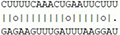
TRINITY_DN14588_c1_g3_i10	osa-miR1435	0.91	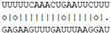
TRINITY_DN12270_c5_g6_i2	ath-miR867	0.86	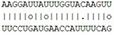
TRINITY_DN14004_c1_g1_i6	ath-miR5021	0.84	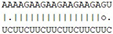
TRINITY_DN14004_c1_g1_i7	ath-miR5021	0.84	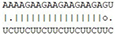
TRINITY_DN13667_c0_g4_i1	osa-miR3979-5p	0.8	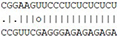
TRINITY_DN14219_c1_g4_i2	ath-miR5021	0.8	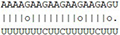
TRINITY_DN14419_c2_g1_i1	*csi*-miR477b	0.79	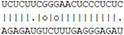
TRINITY_DN14419_c2_g1_i1	mes-miR477d	0.79	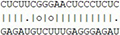
TRINITY_DN14212_c1_g8_i1	ath-miR5021	0.77	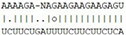
TRINITY_DN11934_c0_g6_i1	ath-miR156i	0.76	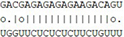
TRINITY_DN11934_c0_g6_i3	ath-miR156i	0.76	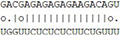
TRINITY_DN13337_c1_g4_i2	ptc-miR169ab	0.74	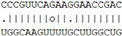
TRINITY_DN14419_c2_g1_i1	cme-miR477b	0.74	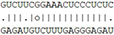
TRINITY_DN11608_c3_g1_i1	ath-miR5021	0.73	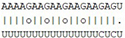
TRINITY_DN13541_c1_g2_i2	mtr-miR5212-5p	0.71	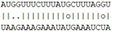
TRINITY_DN13984_c2_g1_i1	mtr-miR5239	0.71	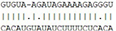
TRINITY_DN14419_c2_g1_i1	mes-miR477f	0.71	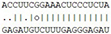
TRINITY_DN14419_c2_g1_i1	mes-miR477g	0.71	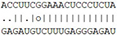
TRINITY_DN14714_c1_g1_i1	ath-miR5021	0.68	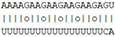
TRINITY_DN11934_c0_g6_i3	*csi*-miR3951	0.68	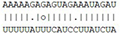
TRINITY_DN11934_c0_g6_i1	*csi*-miR3951	0.68	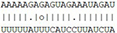

These miRNA targets abundantly belonged to the class of genes associated with cell wall stability and degradation, *namely*, polygalacturonase, polygalacturonase-1 non-catalytic subunit beta, alpha-expansin-3, alpha-expansin-1, and expansin-S1, pectin esterase and beta galactosidase. Besides, trehalose-6-phosphate, heat shock proteins, STS14 protein, Squamosa promoter-binding protein, ethylene responsive, and ACC oxidase were also identified which are key to the ripening process and post-harvest degradation of fruits.

### Identification of mRNA targets of DElncRNAs

Both, the *cis* and *trans* targets of DElncRNAs were identified. In the case of *cis* targets, 1124 unique target genes were found to be targets of 58 DElncRNAs out of 69 DElncRNAs. The remaining 11 DElncRNAs could not be matched to the reference genome. In the case of *trans*-targets, lncTAR was used to identify the mRNA targets of differentially expressed lncRNA where 2,616 unique target genes were found to be interacting with the DElncRNAs (Supplementary Table 8).

The lncRNAs with possible targets which are associated with shelf-life were identified. The DElncRNAs, TRINITY_DN10719_c0_g2_i2 was downregulated in the set 1A:2A and important target genes were Csa_6G363610: extensin-like, Csa_7G322600: extensin-like isoform X1, Csa_7G312940: extensin-3, and Csa_4G017050, Csa_4G291360: extension-2 like. TRINITY_DN10873_c0_g1_i14 was another identified downregulated DElncRNA for the set 1B:2B with targets for the genes associated with chlorophyll biosynthesis and ethylene responsive transcription factors. TRINITY_DN11625_c4_g1_i1 was another important DElncRNA that was down regulated in 1B:2B and targeted the genes associated with cell wall degradation like cell wall/vacuolar inhibitor of fructosidase 2 and Fimbrin-2. The downregulated lncRNAs in the combination 1B:2B were found to target genes associated with probable xyloglucan glycosyltransferase 6 which is closely associated with cell wall degradation. The DElncRNA, TRINITY_DN14508_c2_g11_i3 was found to be downregulated, both in the combinations of 1A:2A and 1B:2B. Xyloglucan glycosyltransferase 6 (Csa_7G341210 and Csa_7G062870) was one of the important target genes of this lncRNA. These results indicated the role of the DElncRNA in determining the shelf life of the cucumber fruits through regulation of the genes associated with cell wall degradation, chlorophyll, and ethylene biosynthesis.

### Identification of circular RNA and differentially expressed circRNA (DEcircRNAs)

**FIGURE 6:**
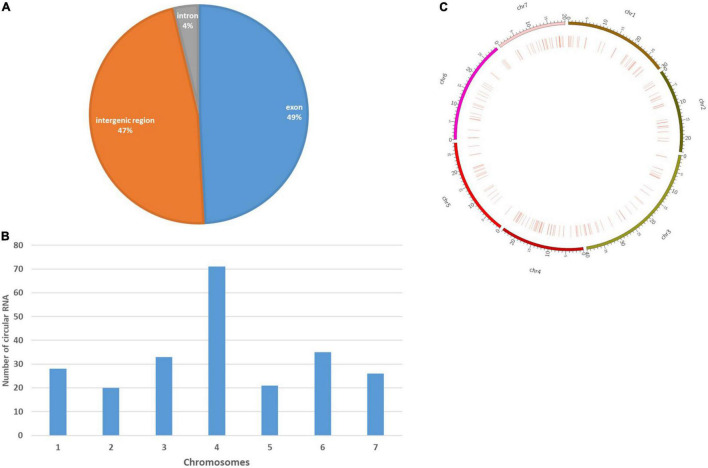
Characterization of cucumber circRNAs. **(A)** Pie chart representing the amount and percentage of circular RNAs generated from exonic, intronic, and intergenic regions. **(B)** Circosplot showing the distribution of circular RNAs in seven cucumber chromosomes. **(C)** Histogram showing the number of circRNAs detected in seven cucumber chromosomes.

CIRI2, the circular RNA (circRNA) identification tool detected 2,746 junction reads from the SAM files. Based on the back-spliced reads, a total of 238 circRNAs were finally detected (Supplementary Table 9). Genomic location analysis of these circRNAs showed that the majority of these identified circRNAs were exonic circRNAs (117, 49%), followed by intergenic circRNAs (112, 47%), and the remaining were intronic circRNAs (9, 4%), hence proving their distribution across the whole genome (Figure 6A). Nevertheless, similar to coding genes, circRNAs were more commonly found to be distributed at both ends of chromosomes (Figure 6B). The chromosome distribution showed an abundance of circRNAs in chromosome 4 (71), followed by chromosomes 6 (35) and 3(33) (Figure 6C). Principal target genes of identified circRNAs were presented in (Supplementary Table 9). Important targets of the identified circRNAs were genes associated with callose synthase (Csa_1G605110, Csa_4G645250), phenylalanine ammonia-lyase (Csa_2G008770), Polyadenylate-binding protein 5 (Csa_3G653460), galactokinase (Csa_3G883000), 4-alpha-glucanotransferase (Csa_4G420150), elongation factor 4 (Csa_4G437010), Probable galacturonosyltransferase 15 (Csa_6G177440), and glutamate formiminotransferase (Csa_6G124080). A total of 2,250 miRNA were identified which could target these circRNAs (Supplementary Table 10). We identified a total of 51 DEcircRNAs, out of which 41 are downregulated while 10 are upregulated under different experimental conditions (Supplementary Table 11). It was also observed a multiple number of circRNAs were involved in regulating a particular gene. It was observed that most of the DEcirc RNAs were detected under the 1B: 2B condition indicating a greater role of these regulatory components at the marketable harvesting stage. One transcript, TRINITY_DN9807_c0_g1_i7 was upregulated under both 1A:2A (11.64) and 1B:2B (8.68) and was found to be regulated by two circRNAs, stout_110 and stout_209. Some of the key transcripts downregulated were TRINITY_DN14188_c4_g4_i3 and TRINITY_DN14188_c4_g3_i2 both of which were regulated by multiple numbers of circRNAs and downregulated at the stage of 1B: 2B.

### Analysis of lncRNA, circRNA, miRNA, and mRNA network interactions

**FIGURE 7:**
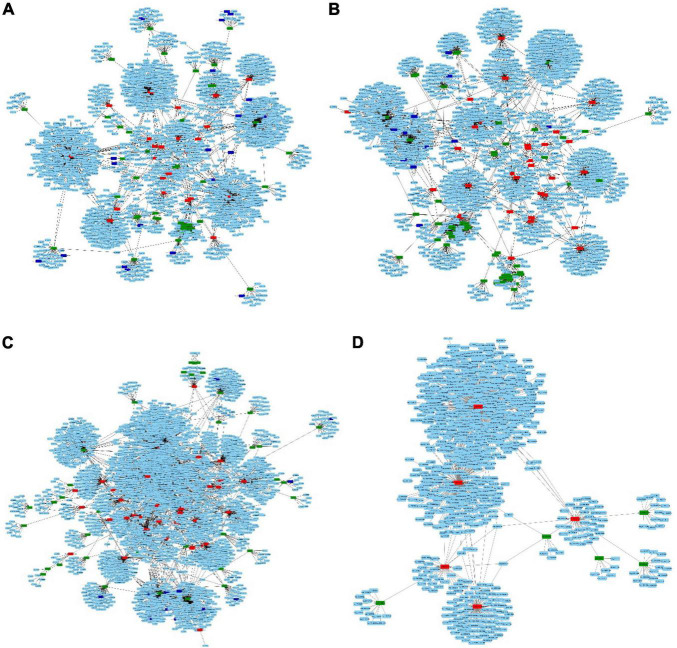
ceRNA network of four comparisons in cucumber. **(A)** 1A1B **(B)** 1A2A **(C)** 1B2B **(D)** 2A2B.

The competing endogenous RNA (ceRNA) network was constructed for each of the four comparison datasets. Nodes represented in green, red, and blue are miRNAs, lncRNAs, and circRNAs while those in light blue are mRNAs (Figures 7A–D). The highest number of interactions in miRNA-lncRNA (86) and miRNA-mRNA (1529) were observed in 1A:2A while 1B:2B had maximum lncRNA-mRNA (2874) and 1A:1B has maximum miRNA-circRNA in the ceRNA network (Table 3 and Supplementary Table 12). It was observed that a multiple number of miRNAs were targeting number of circ RNAs (Supplementary Table 9). The circRNA, stout_120#7:13224577| 13227148 was recorded to be one of the most common targets of miRNAs as a large number of miRNAs were found to be targeting this circRNA. Interactions in miRNA-lncRNA and miRNA-mRNA revealed that a large number of genes are regulated by the ncRNAs and several genes regulated by the network interactions were associated with fruit ripening, cell wall stability, and integrity, ethylene biosynthesis and chlorophyll synthesis, and degradation pathways.

**TABLE 3:** Number of interactions in the ceRNA network.

Comparison	miRNA-lncRNA	miRNA-mRNA	lncRNA-mRNA	miRNA-circRNA
1A1B	47	973	1388	26
1A2A	86	1529	1068	21
1B2B	54	1057	2874	18
2A2B	5	54	965	0

**FIGURE 8:**
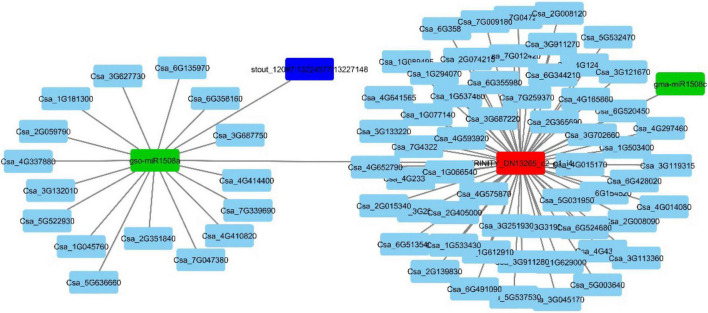
ceRNA network of the validated DElncRNA.

For further detailed insight, the ceRNA network for one of the experimentally validated DElncRNA was constructed. Transcript id TRINITY_DN13265_c2_g1_i4 was differentially expressed under the conditions 1A:1B and 1A:2A (Figure 8).

### Quantitative real-time PCR validation of identified lncRNAs

A total of 17 random transcripts were selected for validation of the RNA-seq data through RT-PCR (Supplementary Table 1). The results indicated a high correlation between the expression pattern of the transcripts between RNA-seq and RT-PCR data for all selected 17 primers (Supplementary Figure 1). Among the 17 selected transcripts, three were down regulated and 14 were upregulated under the different conditions as revealed through RNA-seq and expression analysis using RT-PCR. The expression pattern of the selected transcripts revealed through RNA-seq and RT PCR were similar to each other with no significant difference. The high reliability of the RNA-seq data was elucidated through a similar pattern of the fold change of the RNA-seq and RT PCR data using the randomly selected primers.

### Development of web genomic resources of cucumber lncRNAs

The lncRNA *C. sativus* extended shelf life database (LncR-CsExSLDb), available freely at http://webtom.cabgrid.res.in/lncrcsexsldb. It has five tabs, namely, Home, lncRNAs, circRNAs, Download, and Contact us. LncR-CsExSLDb is a user-friendly and can be easily navigated for browsing the data. The “Download” tab provides links to download all the analyzed data available in the MySQL database. This database catalogs a total of 22,071 predicted lncRNA from cucumber. Out of these, 69 lncRNAs have been identified as differentially expressed lncRNAs (DElncRNAs). It also catalogs 99 miRNAs that can target the identified DElncRNA and these miRNAs in turn can also target 1,228 mRNAs. A total of 3,049 mRNAs were identified as putative targets of lncRNA. Also, a total of 238 circular RNAs are identified which may be the possible targets of 2,250 miRNAs.

## Discussion

Advancement in high-throughput RNA-seq, combined with suitable computational and bioinformatics approaches has revolutionized the discovery of novel lncRNAs and their functional analysis in the last decade (Wang et al., 2009; Iyer et al., 2015). Genes associated with post-harvest biology and the decay of fruits are regulated at transcriptional and post-transcriptional levels. LncRNAs have emerged as one of the important groups regulating the shelf-life of the fruits in different crops (Tian et al., 2019). LncRNAs in plants are associated with numerous biological processes including fruit development, ripening, gene silencing, regulation of flowering time, and abiotic and biotic stress response, besides several other developmental pathways. The natural variant of cucumber, DC-48 with extended shelf-life is an ideal candidate genotype to elucidate the detailed molecular network including the role of lncRNAs and circRNAs in determining the post-harvest biology of the fruits. Insight into the molecular mechanisms participating in gene regulation for enhanced shelf-life in a natural variant of cucumber, DC-48 will provide the much-needed information for further studies on the genotypes with extended shelf-life.

We found the occurrence of the majority of identified lncRNAs having sequence lengths 201-400 bp across the cucumber genome. A similar trend has been observed in grapes (Bhatia et al., 2019). Besides, the genomic locations of lncRNAs were found to be distributed unevenly, but across all the chromosomes. Such chromosomal distribution has been reported in cucumber, grapes, and tomato lncRNAs (Hao et al., 2015; Wang et al., 2015; Bhatia et al., 2019) supporting that they might get transcribed from wider locations in the genome.

A complex network of physiological and metabolic activities is involved in the process of fruit ripening. The role of the lncRNAs in the ripening of both climacteric and non-climacteric fruits has been demonstrated in tomatoes (Zhu et al., 2015), strawberries (Wang et al., 2017), pear (Wu et al., 2014), and melons (Tian et al., 2019). In recent times, several evidence are emerging elucidating the key role of the lncRNAs in the ripening of different groups of fruit crops (Tang et al., 2021). Knocking down of lncRNA1459 and lncRNA1840 in tomatoes resulted in delayed fruit ripening (Zhu et al., 2015). Similarly, in sea buckthorn *in vivo* anthocyanin biosynthesis during fruit ripening was affected by the silencing of two lncRNAs (LNC1 and LNC2) (Zhang et al., 2018). Several lncRNAs associated with different stages of flower and fruit development have been identified in non-climacteric fruit like strawberries through comparative transcriptomic studies (Kang and Liu, 2015). One lncRNA, FRILAIR has been identified recently in strawberries with a key role in fruit ripening by functioning as a non-canonical target mimic (Tang et al., 2021).

LncRNA can act as endogenous target mimics of miRNA, resulting in the blocking of the interaction between miRNA and its target gene (Karakülah et al., 2016). The interaction between the miRNAs and their target genes plays a pivotal role in regulatory gene networks in a wide variety of plants (Meng et al., 2021). Prediction through psRNATarget server revealed that 8183 unique miRNAs were targeting the identified lncRNAs. Similar interaction has been reported in Chinese cabbage under heat stress (Wang et al., 2019), *Arabidopsis* under phosphate deficiency (Franco-Zorrilla et al., 2007), *C. melo* under powdery mildew infestation (Gao et al., 2020), and *C. sativus* under waterlogging condition (Kȩska et al., 2021). Among the different targets, the important genes were extensin like, polygalactoronase-1 non-catalytic sub-unit beta, WRKY domain class transcription factor, xylogucan specific endonuclease inhibitors, Endo 1,4 beta glucanase and Pectinestarase that determine the cell wall stability and fruit firmness. Besides, the genes related to the ethylene metabolism like 1-aminocyclopropane-1-carboxylic acid oxidase, ethylene responsive transcription factors and ethylene receptor CS-ETR 2 were also targeted by multiple miRNAs. Different classes of heat shock proteins (HSPs) namely Cytosolic class-II low molecular weight HSPs, Chloroplast small HSP class-I, HSP-70, and mitochondrial small HSPs were the principal targets. Trehalose-6-phosphate synthase, Squamosa promoter binding protein, WRKY domain transcription factors, MADS box proteins, and abscisic stress ripening inhibitors were other identified miRNA targets. The miRNA target genes, *namely*, trehalose-6-phosphate, heat shock proteins, STS14 protein, squamosa promoter-binding protein, ethylene responsive, ACC oxidase were also identified in our study which are known as a key to the ripening process and post-harvest degradation of fruits (Liu et al., 2015; Upadhyay et al., 2020; Xu et al., 2020). The results revealed the miRNA targets to be associated with genes like polygalacturonase family, alpha-expansin-3, alpha-expansin-1 and expansin-S1, pectin esterase, and beta galactosidase which play important roles in cell wall stability and degradation (Bordenave, 1996; Kalunke et al., 2015; Yang et al., 2018; Valenzuela-Riffo et al., 2020).

It was reported that reduction in polygalacturonase beta subunit expression in tomatoes affects pectin solubilization and degradation of tomato fruits during ripening (Watson et al., 1994). The role of the beta subunit of polygalacturonase-1 (PG-1) in fruit firmness and ripening has been described by Yang et al. (2018). In the present study, it was found that a multiple number of miRNA target genes were associated with the beta subunit of PG-1 which might have a possible role in the retention of fruit firmness and extended shelf-life in the genotype DC-48. Xyloglucan endonuclease, endo 1,4 glucanase, and pectin esterase were reported to play a key role in the stability of cell wall, retention of fruit firmness and regulates the ripening in strawberries and pears (Paniagua et al., 2014; Yang et al., 2016; Witasari et al., 2019). It is well established that WRKY transcription factors (TFs) play important roles in stress responses in different plant species. Recently, the role of of WRKY TFs in fruit ripening and color change has been reported in tomatoes (Wang et al., 2017). It was found that SQUAMOSA promoter binding protein-like transcription factors were a major player in the development and ripening of the papaya fruits (Xu et al., 2020), and this group of transcription factors was found widely distributed among the miRNA target genes in the present study. The trehalose-6-phosphate function is mediated through the non-fermenting-related kinase-1 (SnRK1) pathway (Figueroa and Lunn, 2016). In cucumber, trehalose-6-phosphate and SnRK1-mediated pathways were found to be involved in fruit setting and further development (Zhang et al., 2015). In addition, the role of the SnRK1 pathways in fruit ripening has been reported in tomatoes and apples (Wang et al., 2012; Yu et al., 2018). Heat shock proteins (HSPs) are known to be ubiquitous and highly conserved in nature and their role in abiotic stress tolerance is well established. Small heat shock proteins (sHSP) attracted attention in recent times because of their role in regulating a wide variety of developmental pathways in plants. Recently, two small HSP genes, SlHSP17.7A and SlHSP17.7B which are localized on Chr.6 and Chr.9 in tomatoes were found to regulate the fruit development and ripening and were upregulated during the transition phase of the fruits from mature green to beaker stage (Upadhyay et al., 2020). A large number of HSPs were identified as miRNA target genes in the present study indicating their possible role in the regulation of the fruit development in two contrasting genotypes for extended shelf-life. In tomatoes, MADS box proteins play important role in the regulation of fruit ripening through ethylene synthesis, ethylene response, and ethylene perception (Yuste-Lisbona et al., 2016). Multiple miRNA target MADS box genes were identified in the present study indicating their possible role in the regulation of ethylene biosynthesis and determining the shelf-life of the cucumber fruits.

Among the 97 DElncRNAs in two different genotypes at two different developmental stages, the highest number of DElncRNAs were identified in the combination, 1B: 2B (DC-48 : DC-83 at 10 days after pollination) followed by 1A : 2A (DC-48 : DC-83 at 5 days after pollination). Besides, 18 DElncRNAs were common among the two contrasting genotypes at 5 days and 10 days after pollination indicating a crucial role of these lncRNAs in determining the extended shelf-life of the cucumber fruits. Among the 18 common lncRNAs in two different developmental stages, 8 were downregulated and 10 were upregulated in under both the developmental stages in two contrasting genotypes. The lncRNAs which were downregulated in the genotype with low shelf-life indicated that these lncRNAs acted as the miRNA decoy resulting in their inhibited expression (Kȩska et al., 2021). Among the eight downregulated lncRNAs, most of them had targets related to the genes associated with fruit firmness and chlorophyll biosynthesis explaining their role in determining shelf-life in cucumbers. Similarly, most of the upregulated lncRNAs at both the developmental stages had miRNA target genes associated with ethylene biosynthesis, cell wall degradation, stability, and fruit ripening explaining their critical role in extended shelf-life in the natural variant, DC-48. The identified DElncRNAs in the present study would be instrumental in understanding the role of lncRNAs in determining shelf life in a wide variety of crops.

In the past few years, the regulatory role of circRNAs in different biological processes has been established in transcriptional and post-transcriptional stages. The role of the circRNAs in their function as microRNA (miRNA) sponges has been reported widely in different animals (Bolha et al., 2017). Circular RNAs are often generated by a non-linear back splicing event between a downstream splice donor and an upstream splice acceptor. The development of circRNA sequence in recent time facilitated the identification of several circRNA in both, plants and animals (Zhu et al., 2019). Unraveling the highly expressed and conserved enhanced the functional impact of non-coding RNAs on the regulation of a wide variety of biological processes (Errichelli et al., 2017). Biogenesis, regulation, and function of circRNAs are less understood in plants than compared in animals (Zhou et al., 2018). The difference in the biogenesis of circRNA in plants has been reported through the characterization of circRNAs in *Oryza sativa* and *Arabidopsis thaliana* (Ye et al., 2015). Different studies have established that the plant circRNAs are conserved in nature with low expression levels. The role of circRNA in the regulation of several biological processes like abiotic stresses has been reported in different plants including cucumber under salt stress conditions (Zhu et al., 2019). In watermelon, the regulatory role of circRNAs in resistance to cucumber green mottle mosaic virus infection has been reported (Sun Y. et al., 2020). The circRNAs associated with the transcripts, TRINITY_DN14188_c4_g4_i3, TRINITY_DN14188_c4_g3_i2 were most commonly reported to be differentially expressed in the genotypes, DC-48 and DC-83 at 10 DAP (1B:2B). Expression of TRINITY_DN14188_c4_g4_i3 transcript was regulated by 27 DEcircRNAs, and expression of TRINITY_DN14188_c4_g3_i2 was regulated by 4 DEcircRNA. The identified circDNAs associated with the regulation of expression of the key transcripts must be involved in determining the shelf-life of the cucumber fruits. Besides, the transcript TRINITY_DN9807_c0_g1_i7 was found to be up-regulated among the contrasting genotypes at 5DAP and 10DAP was regulated by the circRNAs stout_110 and stout_209. Our study identified numbers of circRNA regulating the functions of key genes associated with shelf-life in cucumbers. Phenylalanine ammonia-lyase is one of the important enzymes associated with fruit ripening in mango (Palafox-Carlos et al., 2014). Galactokinase was reported to play important role in cell wall stability in the ripening process of yellow melon (Schemberger et al., 2020). It was further established that circRNAs were involved in determining the shelf-life of cucumber fruits in the natural variant, DC-48 with exceptionally high shelf-life and minimum post-harvest degradation through the regulation of the important genes associated with fruit ripening and cell wall stability.

In the present study, it was observed that the majority of the circRNAs were exonic in origin followed by intergenic and intronic types, indicating genome-wide distribution of the circRNAs in cucumber. In the earlier study in cucumber, genome-wide distribution of circRNAs and similar types were reported by Zhu et al. (2019) while studying their regulatory role in salt stress tolerance. Besides, it was also found that circRNAs were also distributed uniformly in both ends of the chromosomes and the highest number of circRNAs were identified in chromosome number 4 followed by chromosomes 6 and 2. Several identified circRNAs in chromosome numbers 4 and 6 were found to regulate the parent genes associated with metabolisms like cell wall stability and degradation, chlorophyll biosynthesis, degeneration, and ethylene biosynthesis. A similar pattern was also observed while studying the target genes associated with the identified DElncRNAs.

The comprehensive web resource of cucumber lncRNAs, LncR-CsExSLDb available to researchers worldwide for the academic purpose in a single place would provide a platform to understand the key roles of lncRNAs, circRNAs, and miRNA targets in the delayed ripening of cucumber. The lncRNA expression may also be used as a promising biomarker for the delayed shelf-life of this very important vegetable crop. The results of this study indicated the regulatory roles of the lncRNAs and circRNAs in determining the shelf-life of the cucumber fruits. These results will be instrumental in the future study of cucumbers in understanding the complex molecular networks and regulatory roles of the ncRNAs in determining complex traits like extended shelf-life in cucumbers. However, further detailed studies need to be conducted to delineate the role of the specific lncRNAs and circRNAs in regulating the specific metabolism associated with extended shelf-life.

## Data availability statement

The original contributions presented in the study are publicly available. This data can be found here: NCBI, PRJNA702645.

## Author contributions

SSD: conceived theme of the study and designed experiment. SSD, MI, and SJ: data curation. PS, MI, SJ, and SSD: computational analysis and development of web-resources. SSD, BG, and KK: investigation. SSD, TKB, and ADM: Resources. SSD, ADM, and RB: supervision. SSD, TKB, ADM, and RB: visualization. SSD, PS, MI, ADM, and SJ: writing original draft. SSD, TKB, AR, and DK: review and editing. All authors read and approved the final manuscript.

## Conflict of interest

The authors declare that the research was conducted in the absence of any commercial or financial relationships that could be construed as a potential conflict of interest.

## Publisher’s note

All claims expressed in this article are solely those of the authors and do not necessarily represent those of their affiliated organizations, or those of the publisher, the editors and the reviewers. Any product that may be evaluated in this article, or claim that may be made by its manufacturer, is not guaranteed or endorsed by the publisher.

## Footnotes

1
http://www.bioinformatics.babraham.ac.uk/projects/fastqc/

2
https://www.ncbi.nlm.nih.gov/orffinder

3
https://hpc.nih.gov/apps/TransDecoder.html

4
https://rnacentral.org

5
http://cantata.amu.edu.pl/

6
http://plncdb.tobaccodb.org/

7
http://ftp.ensemblgenomes.org/pub/plants/release-48/fasta/cucumis_sativus/cdna/

8
https://www.idtdna.com/

9
http://ftp.gramene.org/CURRENT_RELEASE/fasta/cucumis_sativus/dna/

## Supplementary material

Supplementary Figure 1 Experession analysis of the selected 17 transcripts through RT-PCT and fold change through RNA-seq.
