# Supplementary figures and images for: Genome wide identification of lncRNAs and circRNAs having regulatory role in fruit shelf life in health crop cucumber (Cucumis sativus L.)
Source: Front Plant Sci. 2022 Aug 3;13:884476. doi: 10.3389/fpls.2022.884476 (PMC9383263; doi:10.3389/fpls.2022.884476)

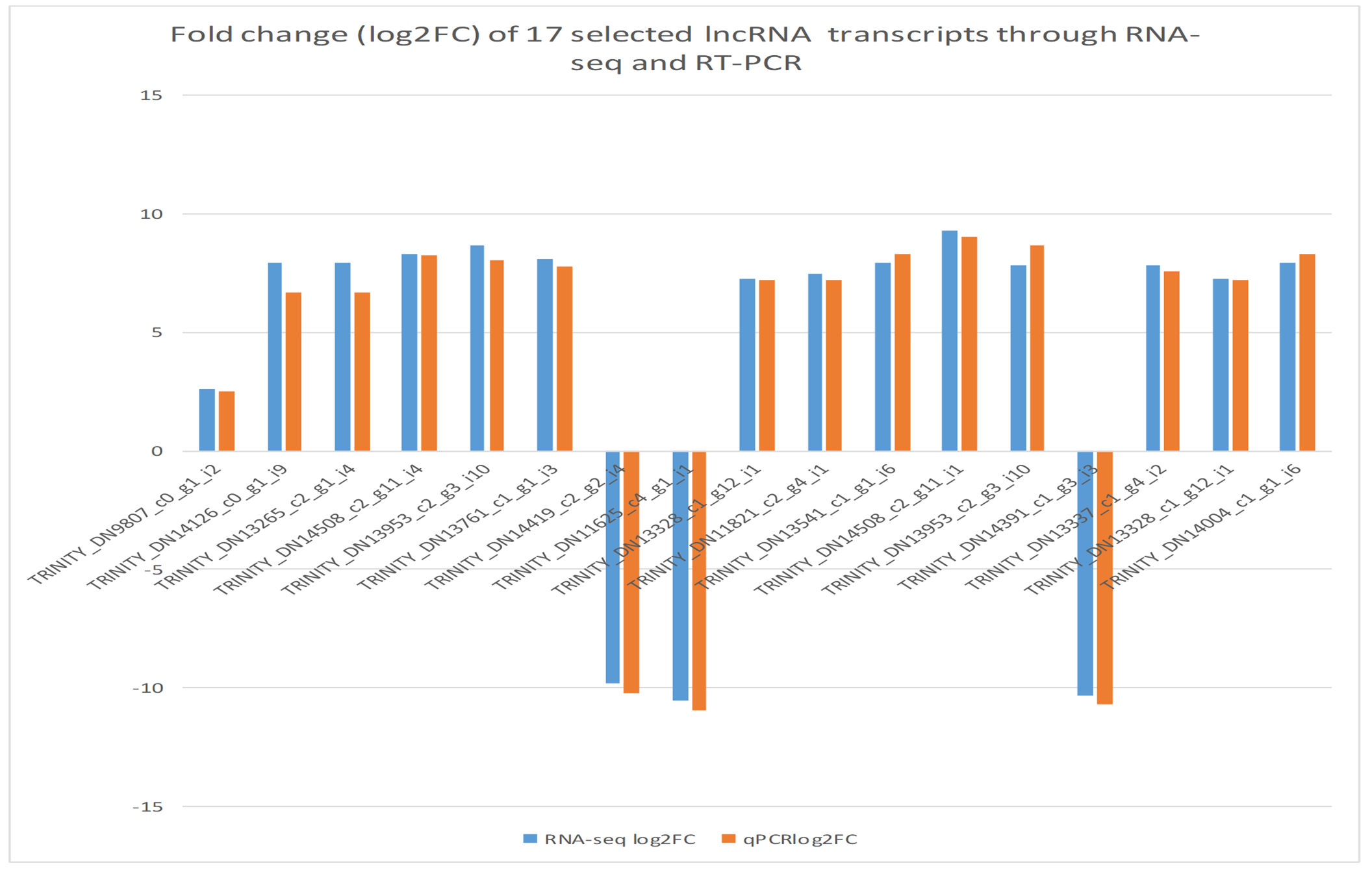

Supplement: Supplementary Figure 1 — Experession analysis of the selected 17 transcripts through RT-PCT and fold change through RNA-seq. [file Image_1.tif]
